# Direct T2 quantification to detect myocardial edema in patients with myocarditis and tako-tsubo cardiomyopathy

**DOI:** 10.1186/1532-429X-13-S1-P313

**Published:** 2011-02-02

**Authors:** Paaladinesh Thavendiranathan, Michael Walls, Shivraman Giri, David Verhaert, Orlando P Simonetti, Subha V Raman

**Affiliations:** 1 The Ohio State University, Columbus, OH, USA

## Background/objective

T2-weighted cardiovascular magnetic resonance (T2W-CMR) has shown utility in diseases such as myocarditis and tako-tsubo cardiomyopathy (TTCM) where myocardial edema may be the predominant pathological abnormality. Since these conditions may affect many myocardial regions, a more quantitative approach may help identify extent of myocardial involvement and reduce uncertainty in qualitative interpretation of T2W-CMR. We sought to evaluate the diagnostic utility of quantitative T2 mapping in patients presenting with acute myocarditis or TTCM.

## Methods

**Figure 1:**
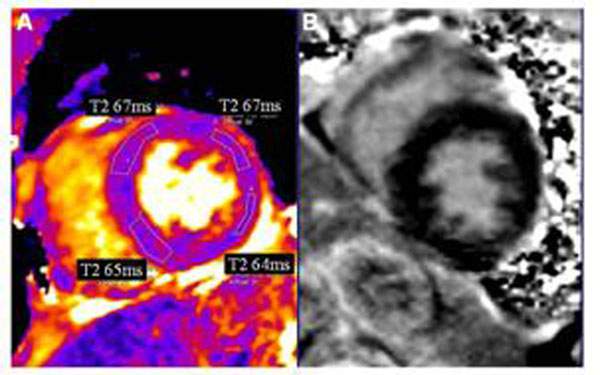
(A) Basal short axis T2 map shows elevated T2 in a patient with myocarditis involving most of the basal segments. (B) Corresponding LGE image shows no hyperenhancement.

Consecutive patients referred for CMR assessment of myocarditis or TTCM who met established diagnostic criteria were enrolled. Healthy volunteers served as controls. Patients’ CMR studies included T2 mapping [1]^)^ on the identical 1.5T scanner with a 12-element phased-array coil (MAGNETOM Avanto). One reviewer blindly scored each patient’s cine and late gadolinium enhancement (LGE) images across 17 myocardial segments. Regions with wall motion abnormality, LGE-positivity and/or visually-apparent T2 map abnormality defined presence of myocardial involvement. Myocardial T2 values were obtained by encircling a region of at least 15 pixels (Figure 1). In controls, T2 values were measured across an entire mid-short axis slice. Abnormal T2 values were defined as exceeding 2SDs above the mean T2 of controls.

## Results

**Figure 2:**
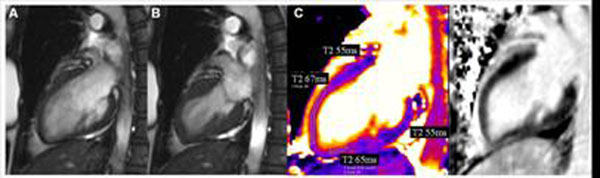
Vertical long axis diastolic (A) and systolic (B) SSFP cinc frames illustrate apical ballooning. (C) T2 map in the same view shows elevated T2 in the apical LV segments and (D) LGE image shows no hyperenhancement.

**Figure 3:**
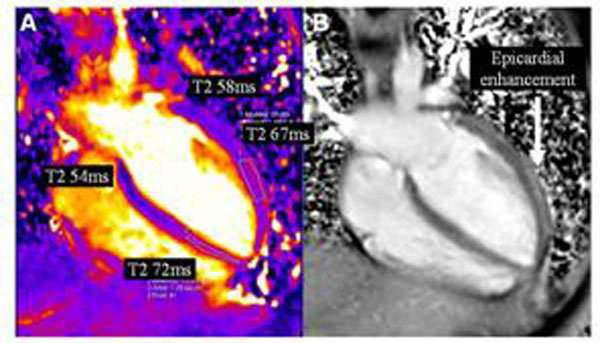
(A) T2 map in a horizontal long axis view in a patient with myocarditis illustrates high T2 values both in the distal septum and lateral wall. (B) Corresponding LGE imaging shows epicardial hyperenhancement online in the lateral wall

Fourteen patients (10 with myocarditis and 4 with TTCM) age 43±17 years and 14 controls were enrolled. Patients’ peak troponin was 13±12ng/ml, time from admission to CMR 1.9±2.3 days, and LVEF 47±9%. T2 maps were successfully obtained in all patients. Patients’ T2 in involved myocardial segments was 66.1±4.0ms vs. 54.9±2.5ms in healthy volunteers’ myocardium (p<0.001). T2 values were similarly elevated in patients with myocarditis and TTCM (65.3±3.4 and 69.0±5.1ms, respectively; p=0.3). No TTCM had abnormal LGE (Figure 2); however, 7/10 patients with myocarditis showed classic LGE abnormalities. Among patients with abnormal LGE, 5 had abnormal T2 values involving 5±2.5 segments beyond the LGE-positive areas (Figure 3).

## Conclusion

T2 quantification identifies myocardial abnormalities in patients with myocarditis and TTCM. Abnormal T2 occurred in the absence of LGE-positivity in all TTCM and 30% of myocarditis patients. In addition, T2 mapping identified areas of edema beyond LGE-positive regions in myocarditis. T2 mapping may provide additional information beyond cine and LGE, suggesting a more comprehensive assessment of myocardial involvement in both myocarditis and TTCM.
